# General Practitioner Management of Shoulder Pain in Comparison with Rheumatologist Expectation of Care and Best Evidence: An Australian National Survey

**DOI:** 10.1371/journal.pone.0061243

**Published:** 2013-04-16

**Authors:** Rachelle Buchbinder, Margaret P. Staples, E. Michael Shanahan, Juliana F. Roos

**Affiliations:** 1 Monash Department of Clinical Epidemiology, Cabrini Health and Department of Epidemiology and Preventive Medicine, School of Public Health and Preventive Medicine, Monash University, Melbourne, Victoria, Australia; 2 Department of Rheumatology, Repatriation General Hospital, Adelaide, South Australia, Australia; Universidade Federal do Acre (Federal University of Acre), Brazil

## Abstract

**Objectives:**

To determine whether current care for common shoulder problems in Australian general practice is in keeping with rheumatologist expectations and the best available evidence.

**Methods:**

We performed a mailed survey of a random sample of 3500 Australian GPs and an online survey of all 270 rheumatologists in Australia in June 2009. Each survey included four vignettes (first presentation of shoulder pain due to rotator cuff tendinopathy, acute rotator cuff tear in a 45 year-old labourer and early and later presentation of adhesive capsulitis). For each vignette, GPs were asked to indicate their management, rheumatologists were asked to indicate appropriate primary care, and we determined best available evidence from relevant Cochrane and other systematic reviews and published guidelines.

**Results:**

Data were available for at least one vignette for 614/3500 (17.5%) GPs and 64 (23.8%) rheumatologists. For first presentation of rotator cuff tendinopathy, 69% and 82% of GPs and 50% and 56% rheumatologists would order a shoulder X-ray and ultrasound respectively (between group comparisons P = 0.004 and P<0001). Only 66% GPs and 60% rheumatologists would refer to an orthopaedic surgeon for the acute rotator cuff tear. For adhesive capsulitis, significantly more rheumatologists recommended treatments of known benefit (e.g. glucocorticoid injection (56% versus 14%, P<0.0001), short course of oral glucocorticoids (36% versus 6%, p<0.0001) and arthrographic distension of the glenohumeral joint (41% versus 19%, P<0.0001).

**Conclusions:**

There is a mismatch between the stated management of common shoulder problems encountered in primary care by GPs, rheumatologist expectations of GP care and the available evidence.

## Introduction

Shoulder pain is a common reason for seeking medical care. The annual prevalence and incidence of care-seeking for shoulder pain is reported to be 2.4% and 1–1.5% respectively [1], [2]. To encourage consistent and optimal care for patients with shoulder disorders, numerous Cochrane and other systematic reviews have synthesised evidence from the research literature [3]–[13], with much of this evidence being incorporated into evidence-based summaries, guidelines and recommendations [14]–[19]. Yet the task of incorporating the latest evidence from research studies into clinical practice remains enormous.

Studies that have examined the extent of uptake of best available evidence into routine GP care for shoulder pain suggest a lack of confidence in making specific diagnoses, and an overreliance on early imaging and early specialist referral [1], [2], [20]–[26]. For example, a 2006 US survey of primary care physicians reported that less than half of those who completed an on-line questionnaire about appropriate management of specific shoulder conditions gave responses consistent with the treatment algorithms devised by the American Academy of Orthopaedic Surgeons (AAOS) [27]. Forty-four percent chose treatment options for adhesive capsulitis consistent with these algorithms, and only 29% chose the recommended management for chronic rotator cuff symptoms.

Knowledge and beliefs, patient expectation, the availability of allied health services, access to investigations and specialists, receipt of continuing medical education (CME) and special interests may all influence uptake of evidence [28], [29]. However the direction of effect may sometimes be unexpected. For example, a recent study noted GPs with a special interest in low back pain were apt to have poorer evidence-based beliefs about back pain and poorer management approaches compared with GPs without a special interest [29].

Recently the Australian National Health & Medical Research Council (NHMRC) have established a Research Translation Faculty tasked with identifying important gaps between research evidence and health policy and practice among 17 major health issues, and recommending to NHMRC the action needed to address the gap [30]. A major health issue in Australia is ‘Arthritis and Musculoskeletal Conditions’, one of nine Australian National Health Priority Areas (NHPAs) [31].

To determine whether there is an important evidence-practice gap for management of common shoulder problems in primary care in Australia, we used GP surveys to determine the current pattern of care for shoulder pain in Australian general practice. We also performed a rheumatologist survey to determine Australian rheumatologist expectations of GP care. Australian rheumatologists consider themselves to be experts in the diagnosis and management of rheumatic and musculoskeletal disorders including all forms of arthritis, connective tissue disease, spinal and soft tissue disorders, chronic musculoskeletal pain syndromes and certain metabolic bone disorders, particularly osteoporosis [32]. Therefore the rheumatologist survey would also identify any important evidence-practice gaps among rheumatologists. Responses from both physician groups were compared to the best available evidence, and the level of agreement between GPs and rheumatologist expectations of GP care were assessed. These data may be of use in developing a case for NHMRC action and determining whether general or more targeted approaches may be needed to bridge any detected evidence-practice gaps.

## Materials and Methods

### GP Survey

We performed a mailed survey of a random sample of Australian GPs identified through the Australasian Medical Publishing Company (AMPCo), the publisher of the Medical Directory of Australia [33] in June 2009. A cover letter explained that the purpose of the study was to understand how GPs currently manage patients with shoulder pain and that the data obtained would be valuable for informing how the management of this common and challenging problem could be improved. To maintain confidentiality AMPCo mailed individually numbered questionnaires to GPs and the questionnaires did not contain identifying information. Several strategies, based upon a modified Dillman technique [34], were used to maximise response rate including use of a respondent-friendly design (coloured background fields with white answer spaces and prominent question numbers); a pre-notice letter explaining the reasons for the survey; and a postcard thank you/reminder 3–4 days after sending the questionnaire. GPs were also advised that returned questionnaires would go into a draw for seven $50 book vouchers.

The GP questionnaire was modified from one developed to assess GP management of back pain in Australia [35],[36]. It included four vignettes modified from a Canadian study assessing GP management of common musculoskeletal problems [20], and the US study described above [24]. The vignettes present a 77 year-old female with a six-week history of shoulder pain consistent with rotator cuff tendinopathy; a 45 year-old labourer with clinical features consistent with an acute rotator cuff tear; a 50 year old female with a three week history compatible with adhesive capsulitis; and the same patient presenting three months later with persistent symptoms (Appendix S1). The response options for the scenarios were listed under categories of investigations, consultation management including advice and medications and referrals, and we asked doctors to indicate any they would routinely advocate. We also asked doctors to indicate the likely prognosis and the likelihood that surgery would be required as either very likely, likely, not sure, unlikely or very unlikely. Demographic details and training, membership of societies and associations, special interests, preferences and sources for keeping up to date, and practice characteristics were also collected. The IDs of seven GPs were drawn at random from all returned questionnaires and $50 book vouchers sent to AMPCo for forwarding to these GPs.

### Rheumatologist Survey

We performed an online survey of all currently practicing rheumatologists in Australia as indicated by their membership of the Australian Rheumatology Association (ARA) in June 2009. Responses were confidential. The ARA approved the survey and sent all clinical members a link to SurveyMonkey. To maximise response rate, reminder emails were sent 2 and 6 weeks later. Respondents could leave their name and contact details separately to go into a draw for a raffle for one of three book vouchers valued at $50 each.

The rheumatologists were presented with the same four vignettes and asked to indicate what management they would routinely advocate in primary care. Demographic details, medical training, preferences for keeping up to date, and practice characteristics were also collected.

The Cabrini Health and Monash University Human Research Ethics Committees approved the study.

### Determination of Best Evidence-based Care

To determine whether participant responses for each of the scenarios were consistent with the best available evidence we compared responses to relevant Cochrane and other systematic reviews and published guidelines [15]–[19], [27], [37]. Two publications were of particular relevance to the Australian context, both written by a multidisciplinary panel and based upon evidence: the NHMRC-endorsed Evidence-based Management of Acute Musculoskeletal Pain Guidelines published in 2003 [15] and Therapeutic Guidelines: Rheumatology version 2 [17]. Therapeutic Guidelines is a widely respected and available resource for GPs in Australia. The first Rheumatology version was released in April 2006 and an update was released in November 2010, both endorsed by multiple organisations including the ARA, The Royal Australian College of General Practitioners and the Australian Physiotherapy Association. Appendix S2 presents a summary of our synthesis of the best available evidence as it applies to the four vignettes.

### Sample Size

We estimated that responses from at least 103 GP and 103 rheumatologists would be required to detect a difference of 20% between the two groups in the proportion of agreeing with the specified options for investigations, management or referral (5% significance level, 80% power). Allowing for around 80% non-response from GPs, we approached a random sample of 3500 to recruit 670 GPs. We invited all 270 practising rheumatologists in Australia to complete the online survey requiring a response rate of 38% to achieve a total of 103 rheumatologists.

### Statistical Analysis

Descriptive statistics were used to summarize the demographics and practice characteristics of study participants. The proportion of responses for the management of each scenario was determined for both the GPs and the rheumatologists. For the prognosis items, we calculated the proportion of respondents who indicated the outcome was either ‘likely’ or ‘very likely’. Comparisons of proportions were analysed with a chi squared (χ^2^) test of independent samples. Because of the multiplicity of comparisons we have reported p-values <0.05 but have commented only on those with a p-value <0.001. Comparisons between doctors with or without recent CME in musculoskeletal disorders were evaluated in the same way. Comparisons between doctors with and without a special interest in musculoskeletal disorders and/or occupational medicine were also planned but were not performed because of too few numbers.

All statistical analyses were performed using Stata/IC 11.0.

## Results

Of the 3500 questionnaires mailed to GPs, 639, including 25 blank forms, were returned. Three GPs did not answer the demographic questions but completed at least one vignette giving a response rate for completing at least one vignette of 17.5% (614/3500). Eighty rheumatologists opened the survey link in the email; one did not see general rheumatology patients and was thus ineligible; nine did not answer any demographic questions or attempt any of the vignettes; the response rate for demographic questions was 26.0% (70/269). Six rheumatologists who completed the demographics did not attempt any of the vignettes. Data were available for at least one vignette from 64 rheumatologists (response rate 23.8%). Those who did not complete any vignettes did not differ from other respondents by gender (p = 0.96), year of graduation (p = 0.20), number of patients seen per week (p = 0.80) or number of patients with shoulder pain per week (p = 0.77).

Demographic and practice characteristics and top preferences for keeping up to date for GPs and rheumatologists are reported in Table 1. For both groups, about two–thirds of respondents were male and median duration of practice was 20 years. Just over a third of GPs had received CME in musculoskeletal disorders in the preceding two years. Journal articles were the top preference for keeping up to date among both GPs and rheumatologists.

**Table 1:** Demographics and characteristics of general practitioners and rheumatologists.

	GPs (N = 611)*	Rheumatologist (N = 70)
Male, N (%)	378 (62)	46 (66)
Year of graduation, median (range)	1981 (1952–2005)	1981 (1963–2000)
Practice location, N (%), N = 608 (>1 possible)		
Metropolitan	361 (59)	58 (83)
Regional	130 (21)	19 (27)
Rural	119 (20)	5 (7)
Missing	3 (<1)	0
Practice type		
Conventional practice	534 (87)	–
Bulk billing/24 hour practice	75 (12)	–
Both	2 (<1)	–
Number of doctors in practice		
Solo	83 (14)	–
2–4	198 (32)	–
5–10	251 (41)	–
>10	79 (13)	–
Training (N = 607) (>1 possible)		
Hospital internship	403 (66)	–
Family medicine program	334 (55)	–
Fellowship, RACGP	60 (10)	–
Obstetrics and gynaecology	26 (4)	–
Diploma of Musculoskeletal medicine	14 (2)	–
Membership of medical societies (>1 possible)		
None specified	127 (21)	–
Australian Medical Association	231 (38)	–
RACGP	406 (67)	–
AAMM	8 (1)	
CME/postgraduate training in musculoskeletal		
medicine in last 2 years	219 (36)	–
Special interest in musculoskeletal disorders	83 (14)	–
Special interest in occupational medicine	36 (6)	–
Number of patients seen per week (N = 606)		
<50	90 (15)	30
50–100	175 (29)	28
101–150	204 (33)	11
151–200	100 (16)	0
>200	37 (6)	0
Median (range) patients seen per week for	(N = 597)	
shoulder pain	3 (0–20)	5 (1–20)
Top preference for keeping up to date (N = 607)		
Journal articles	252 (42)	35 (50)
Hospital meetings	–	13 (19)
Conferences	102 (17)	15 (22) #
College (e.g., RACGP) sponsored courses	100 (17)	–
Drug company sponsored meetings	97 (16)	0
Cochrane reviews	12 (2)	1 (1)
∧Other resources	46 (8)	6 (1)

RACGP Royal Australasian College of General Practice, AAMM Australian Association of Musculoskeletal Medicine.

* 613 GPs provided responses for at least one vignette but 2 GPs did not complete any demographic items.

# International conference N = 9 (13%) and Australian conference N = 6 (9%).

∧ GPs: most commonly online resources such as *UpToDate*, specialists and local meetings’ rheumatologists: most commonly *UpToDate.*

### Vignette 1: Rotator Cuff Tendinopathy

Tables 2, 3, 4 5 shows the management responses selected by GPs and rheumatologists for each of the vignettes. While the best available evidence suggests that in the absence of red flags no tests are necessary (see Appendix S1), only 10% of GPs and 34% of rheumatologists indicated no tests were necessary (between-group difference p<0.0001)(Table 2). The majority of GPs would order a shoulder X-ray (69%) and/or an ultrasound (82%) while at least half of the rheumatologists indicated shoulder X-ray and/or ultrasound should be performed.

**Table 2:** GP and rheumatologist management for Vignette 1 (Rotator cuff tendinopathy).

	General	Rheumatologists	P-value
	Practitioners		
	(N = 613)	(N = 64)	
	N (%)	N (%)	
**Investigations**			
Would not order any tests	59 (10)	22 (34)	<0.0001
X-ray	421 (69)	32 (50)	0.004
Ultrasound	503 (82)	36 (56)	<0.0001
CT scan	4 (<1)	0	
MRI scan	7 (1)	1 (2)	
Bone scan	6 (1)	1 (2)	
Blood tests (e.g., FBE, ESR)	141 (23)	11 (17)	
**Consultation management/advice**			
Expectant observation only	58 (10)	6 (9)	
Advice on home exercise	366 (60)	54 (84)	<0.0002
Activity/work modification	346 (56)	41 (64)	
Psychosocial evaluation	75 (12)	6 (9)	
Ice or heat	153 (25)	13 (20)	
Immobilise in sling	3 (<1)	1 (2)	
Prescribe medication	387 (63)	39 (61)	
Over-the-counter analgesics	352 (57)	35 55)	
Prescription analgesics	137 (22)	11 (17)	
Prescription NSAIDS	194 (32)	24 (38)	
Short course of oral glucocorticoids	19 (3)	4 (6)	
Benzodiazepines	6 (1)	0	
Low dose anti-depressants	27 (4)	4 (6)	
Administer a glucocorticoid injection	172 (28)	39 (61)	<0.0001
∧Subacromial space	208 (34)	44 (69)	<0.0001
∧Glenohumeral joint	15 (2)	2 (3)	
∧AC joint	10 (2)	2 (3)	
∧Area of maximal tenderness	51 (8)	0	0.03
Refer for image-guided glucocorticoid			
injection	145 (24)	21 (33)	
Refer for arthrographic distension of			
the glenohumeral joint (hydrodilatation)	9 (2)	1 (2)	
Administer or refer for suprascapular			
nerve block	1 (<1)	0	
**Referral**			
Would not refer	88 (14)	8 (13)	
Physiotherapy	455 (74)	49 (77)	
Chiropractor	6 (1)	0	
Acupuncture	41 (7)	2 (3)	
Orthopaedic surgeon	89 (15)	7 (11)	
Rheumatologist	29 (5)	26 (41)	<0.0001
**Prognosis - Likely or very likely to:**			
Recover within 2 weeks	87 (14)	17 (27)	
Recover within 6 weeks	348 (57)	46 (72)	
Recover within 1–2 years	454 (74)	53 (83)	
Have a recurrence within 2 years	336 (65)	44 (69)	
Have permanent difficulties with			
activities of daily life	123 (20)	3 (5)	0.001
Require surgery	36 (6)	2 (3)	

∧ Some people indicated where they would place the glucocorticoid injection even though they did not indicate that they would administer the injection.

**Table 3:** GP and rheumatologist management for Vignette 2 (Acute rotator cuff tear).

	General	Rheumatologists	P-value
	Practitioners		
	(N = 609)	(N = 59)	
	N (%)	N (%)	
**Investigations**			
Would not order any tests	11 (2)	6 (10)	0.0005
X-ray	163 (27)	19 (32)	
Ultrasound	573 (94)	53 (90)	
CT scan	39(6)	2 (3)	
MRI scan	98 (16)	8 (14)	
Bone scan	4 (1)	1 (2)	
Blood tests (e.g., FBE, ESR)	12 (2)	1 (2)	
**Consultation management/advice**			
Expectant observation only	32 (5)	1(2)	
Advice on home exercise	185 (30)	23 (39)	
Activity/work modification	384 (63)	48 (81)	0.006
Psychosocial evaluation	82 (13)	11 (19)	
Ice or heat	144(24)	16, (27)	
Immobilise in sling	40 (7)	2 (3)	
Prescribe medication	478 (78)	48 (81)	
Over-the-counter analgesics	339 (55)	33 (56)	
Prescription analgesics	201 (33)	23 (39)	
Prescription NSAIDS	331 (54)	39(66)	
Short course of oral corticosteroids	9 (2)	0	
Benzodiazepines	8 (1)	4 (7)	
Low dose anti-depressants	201 (33)	23 (39)	
Administer a glucocorticoid injection	36 (6)	10 (17)	0.004
∧Subacromial space	47 (8)	11 (19)	
∧Glenohumeral joint	6 (1)	1(2)	
∧AC joint	9(2)	1 (2)	
∧Area of maximal tenderness	23 (4)	1 (2)	
Refer for image-guided glucocorticoid			
injection	62(10)	7 (12)	
Refer for arthrographic distension of the			
glenohumeral joint (hydrodilatation)	5 (1)	0	
Administer or refer for suprascapular nerve			
block	0	0	
**Referral**			
Would not refer	16 (3)	4 (7)	
Physiotherapy	372(61)	30 (51)	
Chiropractor	1 (0)	0	
Acupuncture	14(2)	1 (2)	
Orthopaedic surgeon	404 (66)	35 (60)	
Rheumatologist	9 (2)	29 (49)	<0.0001
**Prognosis - Likely or very likely to:**			
Recover within 2 weeks	39 (8)	6 (10)	
Recover within 6 weeks	211 (39)	16 (27)	
Recover within 1–2 years	406 (80)	43 (73)	
Have a recurrence within 2 years	108 (22)	13 (22)	
Have permanent difficulties with activities			
of daily life	84 (16)	9 (15)	
Require surgery	321 (58)	29 (49)	

∧ Some people indicated where they would place the glucocorticoid injection even though they did not indicate that they would administer the injection.

**Table 4:** GP and rheumatologist management for Vignette 3 (Adhesive capsulitis, symptoms for 3 weeks).

	General	Rheumatologists	P-value
	Practitioners		
	(N = 612)	(N = 59)	
	N (%)	N (%)	
**Investigations**			
Would not order any tests	29 (5)	9 (15)	0.002
X-ray	449 (73)	42 (71)	
Ultrasound	459 (74)	34 (58)	0.007
CT scan	38 (6)	2 (3)	
MRI scan	32 (5)	2 (3)	
Bone scan	36 (6)	3 (5)	
Blood tests (eg. FEB, ESR)	279 (46)	29 (49)	
Blood glucose	35 (6)	10 (17)	0.002
**Consultation management/advice**			
Expectant observation only	52 (9)	2 (3)	
Advice on home exercise	260 (42)	42 (71)	<0.0001
Activity/work modification	320 (52)	38 (64)	
Psychosocial evaluation	99 (16)	6 (10)	
Ice or heat	130 (21)	11 (19)	
Immobilise in sling	43 (7)	0	
Prescribe medication	508 (83)	46 (78)	
Over-the-counter analgesics	313 (51)	29 (49)	
Prescription analgesics	310 (51)	28 (48)	
Prescription NSAIDS	380 (62)	29 (49)	0.05
Short course of oral glucocorticoids	39 (6)	21 (36)	<0.0001
Benzodiazepines	25 (4)	0	
Low dose anti-depressants	69 (11)	8 (14)	
Administer a glucocorticoid injection	88 (14)	33 (56)	<0.0001
∧Subacromial space	61 (10)	12 (20)	0.02
∧Glenohumeral joint	80 (13)	33 (56)	<0.0001
∧AC joint	6 (1)	0	
∧Area of maximal tenderness	17 (3)	0	
Refer for image-guided glucocorticoid			
injection	87 (14)	15 (25)	<0.0001
Refer for arthrographic distension of the			
glenohumeral joint (hydrodilatation)	79 (13)	10 (17)	
Administer or refer for suprascapular			
Nerve block	5 (1)	6 (10)	<0.0001
**Referral**			
Would not refer	46 (8)	4 (7)	
Physiotherapy	402 (66)	38 (64)	
Chiropractor	5 (1)	0	
Acupuncture	26 (4)	3 (5)	
Orthopaedic surgeon	170 (28)	8 (14)	0.03
Rheumatologist	107 (18)	44 (75)	<0.0001
**Prognosis - Likely or very likely to:**			
Recover within 2 weeks	30 (7)	0	
Recover within 6 weeks	127 (23)	1 (2)	<0.0001
Recover within 1–2 years	444 (80)	54 (92)	0.03
Have a recurrence within 2 years	151 (30)	7 (12)	0.004
Have permanent difficulties with			
activities of daily life	114 (22)	11 (19)	
Require surgery	51 (10)	0	0.01

∧ Some people indicated where they would place the glucocorticoid injection even though they did not indicate that they would administer the injection.

**Table 5:** GP and rheumatologist management for Vignette 4 (Adhesive capsulitis, same patient as Vignette 3 two months later).

	General	Rheumatologists	P-value
	Practitioners		
	N = 606)	N = (59)	
	N (%)	N (%)	
**Investigations**			
Would not order any tests	191 (31)	34 (59)	<0.0001
X-ray	90 (15)	15 (26)	0.04
Ultrasound	178 (29)	16 (28)	
CT scan	85 (14)	1 (2)	0.01
MRI scan	125 (20)	4 (7)	0.02
Bone scan	50 (8)	2 (3)	
Blood tests (eg. FEB, ESR)	140 (23)	9 (16)	
Blood glucose	10 (2)	3 (5)	
**Consultation management/advice**			
Expectant observation only	60 (10)	4 (7)	
Advice on home exercise	268 (44)	37 (64)	0.0005
Activity/work modification	263 (43)	30 (52)	
Psychosocial evaluation	128 (21)	6 (10)	
Ice or heat	72 (12)	3 (5)	
Immobilise in sling	2 (<1)	0	
Prescribe medication	289 (47)	26 (45)	
Over-the-counter analgesics	258 (42)	23 (40)	
Prescription analgesics	180 (29)	17 (29)	
Prescription NSAIDS	251 (41)	23 (40)	
Short course of oral corticosteroids	23 (4)	4 (7)	
Benzodiazepines	10 (2)	0	
Low dose anti-depressants	90 (15)	8 (14)	
Administer a glucocorticoid injection	81 (13)	16 (28)	
∧Subacromial space	58 (10)	5 (9)	
∧Glenohumeral joint	78 (13)	18 (31)	0.0003
∧AC joint	5 (1)	0	
∧Area of maximal tenderness	29 (5)	0	
Refer for image-guided glucocorticoid			
injection	106 (17)	6 (10)	
Refer for arthrographic distension of the			
glenohumeral joint (hydrodilatation)	115 (19)	24 (41)	0.0001
Administer or refer for suprascapular			
nerve block	3 (1)	4 (7)	0.0001
**Referral**			
Would not refer	30 (5)	2 (3)	
Physiotherapy	350 (57)	36 (62)	
Chiropractor	6 (1)	0	
Acupuncture	33 (5)	3 (5)	
Orthopaedic surgeon	299 (49)	11 (19)	<0.0001
Rheumatologist	128 (21)	42 (72)	<0.0001
**Prognosis - Likely or very likely to:**			
Recover within 2 weeks	18 (4)	0 (0)	
Recover within 6 weeks	76 (15)	1 (2)	0.006
Recover within 1–2 years	445 (79)	50 (86)	
Have a recurrence within 2 years	162 (32)	13 (22)	
Have permanent difficulties with			
activities of daily life	167 (32)	16 (28)	
Require surgery	96 (19)	2 (3)	0.004

∧ Some people indicated where they would place the glucocorticoid injection even though they did not indicate that they would administer the injection.

Both GPs and rheumatologists were broadly consistent with Australian recommendations [17] to provide advice on activity, work modification and home exercise and about three-quarters of both groups would refer for physiotherapy. A third of both groups would prescribe NSAIDs despite the patient having failed a previous 2-week course. Over half the GPs recommended glucocorticoid injection, as did nearly all the rheumatologists (94%). However just under a quarter of GPs and a third of rheumatologists indicated this should be guided by imaging. While there is evidence for the short-term benefit of glucocorticoid injection, image guidance does not appear to provide added benefits (see Appendix S2). Appropriately, only a minority of GPs indicated they would refer to an orthopaedic surgeon or rheumatologist although 41% of rheumatologists indicated referral to rheumatology was appropriate (vs 5% GPs, P<0001).

### Vignette 2: Acute Rotator Cuff Tear

Most GPs and rheumatologists appropriately indicated that further imaging and orthopaedic referral was required (Table 3, Appendix S2), and approximately half indicated that surgery was likely. Similar to Vignette 1, only 2% of GPs versus 49% of rheumatologists advocated referral to a rheumatologist (P<0.0001). A third of GPs and 39% of rheumatologists indicated they would prescribe low-dose antidepressants for this vignette. While anti-depressants are widely used to treat chronic painful musculoskeletal conditions [38], it is unclear what the rationale, if any, would be for acute pain.

### Vignettes 3 and 4: Early and Late Presentation of Adhesive Capsulitis

Similar to vignette 1, the majority of GPs and rheumatologists would also routinely order X-rays (73% and 71% respectively) and ultrasound (74% and 58% respectively) for Vignette 3 S2). Almost half of both groups would order blood tests but only a minority specified blood glucose measurement, which would be a reasonable decision as diabetes is a known risk factor for this condition [39].

Significantly more rheumatologists recommended treatments of known benefit including glucocorticoid injection (56% versus 14% GPs, P<0.0001), a short course of oral glucocorticoids (36% versus 6% of GPs, P<0.0001), home exercises (71% vs 42% for vignette 3, P<0.0001), and referral for arthrographic distension of the glenohumeral joint for the later presentation (41% versus 19% of GPs, P<0.0001). Similar to the other vignettes, significantly less GPs would refer the patient to a rheumatologist for vignettes 3 and 4 and significantly more would refer to orthopaedics.

The majority of GPs and rheumatologists indicated that recovery was likely or very likely to occur within one to two years in both vignettes 3 and 4 in keeping with the reported favourable course of adhesive capsulitis [40]–[42]. However a third of GPs and a quarter of rheumatologists erroneously believed that recurrence was either likely or very likely within 2 years although recurrences have not been reported [40]–[42].

There were no differences between doctors who had or had not completed CME or postgraduate education about musculoskeletal disorders in the past two years (data not shown).

## Discussion

Based upon national surveys of GPs, the current pattern of care in Australian general practice for four common presentations of shoulder pain is highly variable and often suboptimal. In general, there was a high reliance on imaging, particularly plain radiographs and ultrasound, by both GPs and rheumatologists. For each vignette there was a wide variation in what treatments GPs would prescribe, what referrals would be made and the expected prognosis. This was often mirrored by a similar degree of variation in responses among rheumatologists and there were significant discrepancies between GP responses and rheumatologist expectation of GP care.

Our finding of a high dependence upon plain radiographs and ultrasound in all scenarios is in keeping with previous studies [20], [21], [25], [43]. Although a Canadian multidisciplinary panel did consider a plain radiograph standard of care for vignette 1 in 1998 [20], no recent guidelines advocate imaging for shoulder pain unless there is a suggestion of serious pathology [15], [17]–[19]. The results of plain radiographs are unlikely to influence GP management and there are risks associated with radiation exposure.

The diagnostic utility of shoulder ultrasound in primary care is unknown yet in Australia there has been a rapid rise in its use [43], [44]. The procedure is highly operator-dependent, asymptomatic changes on ultrasonography are common and increase with age [45], and many observed abnormalities might not require specific treatment [44]. This has led to concerns that ultrasound findings might be misleading and result in inappropriate and/or delay correct diagnosis and management [17]. One study that reviewed a set of ultrasound request forms found that the motivation for the request was often unclear with a third containing no contributory information for the radiologist [43].

Concomitant with the rise in diagnostic ultrasound for shoulder pain, referral for image-guided injections has risen dramatically in the last decade [44]. Although the exact number of ultrasound-guided shoulder injections being performed in Australia is unknown as the Medicare item number includes other regions of the body, there has been a more than 34-fold increase in the number of ultrasound-guided injections billed to Medicare in the last ten years (3504 per 100,000 population in the 2000–2001 financial year to just over 120,000 per 100,000 population in the 2010–2011 financial year) [46]. Interpretation of this data is also hampered by the fact that government subsidy of anatomically guided joint injections was discontinued in November 2009 which may explain some of the increased use of image-guided injections.

Our finding of a high reliance upon specialist referral is in keeping with some [24], [47], but not all [1], [48], studies. Although many rheumatologists in our study considered specialist referral appropriate for new presentation of non-traumatic shoulder pain, a lack of GP confidence to diagnose and manage common shoulder complaints may lead to unwarranted or premature specialist referrals [47], [49]. For example, a national UK study found that patients presenting with shoulder complaints to general practice are not assigned a specific diagnosis [1]. A recent Australian study also found that only 40% of GP referral letters to an orthopaedic clinic offered a diagnosis [25]. GPs and rheumatologists in our study also differed markedly with respect to preferences for specialist referral. This is in keeping with a 1985 US study that found that GPs regarded orthopaedic surgeons more effective than rheumatologists in treating acute shoulder pain and shoulder tendinitis [50].

Despite the availability of research evidence to inform clinical practice for shoulder pain in primary care, our study indicates an ‘evidence-practice’ gap among both GPs and rheumatologists. Interventions are needed to improve diagnosis and treatment of common shoulder problems in primary care and to reduce inappropriate and costly imaging and specialist referral. Several interventions to change physician management of shoulder pain have shown promising results. Academic detailing has shown favourable results with respect to reducing imaging requests and improving GP knowledge and confidence in managing shoulder problems in one before-after study [51]. A randomised controlled trial found that provision of practical training that included injection techniques to GPs did not improve patient shoulder-specific disability or pain at one year when compared to no training [52]. Another non-randomised controlled trial found that a short skills training course that included shoulder injection technique improved scores on a 5-item knowledge test 3 months after the training and increased use of glucocorticoid injection [53]. Joint consultations with GPs and orthopaedic surgeons were found to reduce referral rates when compared to usual care in a randomised controlled trial [54]. Finally, the development and dissemination of local referral guidelines to a musculoskeletal service in the UK appeared to improve appropriate referrals and reduce waiting times [49].

The strengths of our study include our robust survey methodology, assurance of de-identified data, and assessment of GP management from the perspective of both Australian rheumatologists and the published literature. On the other hand, we measured stated rather than actual practice. However while the relationship between behavioural simulation and direct measures of clinician behaviour is not perfect [55], [56], there is some evidence to support the use of vignettes as a proxy measure for clinician behaviour [57]. While patient preference and/or perceived patient pressure are known to be important influences in clinical practice [58], this may have been less likely to influence stated management responses to paper vignettes.

Furthermore, the validity of our findings is supported by the demonstrably high use of imaging and image-guided injection for shoulder pain in Australia and its consistency with studies performed elsewhere. Some of the variation in GP and rheumatologist responses we observed could be related to local and/or regional set up of services and access to specialists. However our findings of a general overreliance on imaging and referral would seem to indicate that lack of access was not really a factor in GP stated responses.

Another potential limitation of our study is that the generalisability of results is limited by our low response rate. Unfortunately due to issues of confidentiality we were unable to determine whether there were major differences between responders and non-responders. However in comparing the demographics of respondents with data from the Australian GP workforce study 2001 to 2004, respondents were of similar sex (males: 62% vs 63% in the Australian GP workforce) but older age (mean: approximately 53 years based upon year of graduation vs 48.6 years in the Australian GP workforce), and a lesser proportion were working in rural settings (19.4% versus 26.6%) [59]. As well, while it is possible that GPs who have had CME in musculoskeletal disorders in the past two years may have been more inclined to complete the survey, we found no substantial differences in management between GPs with and without a history of CME.

Another limitation may have been our decision, in keeping with other studies [20], [24], [60], not to ask respondents to stipulate their diagnosis. It is therefore not possible to separate diagnostic from management acumen although it is likely that these skills are highly interdependent. For example, the low uptake of both intra-articular and oral glucocorticoids among GPs for adhesive capsulitis may be a result of misdiagnosis of the vignette, lack of awareness of these treatment approaches, lack of confidence in injection techniques and/or concern about potential harms. It would also be of relevance to obtain the perspective of orthopaedic surgeons.

## Conclusion

In summary, there is a mismatch between the stated management of common shoulder problems encountered in primary care by GPs, rheumatologist expectations of GP care and the available evidence. Our results indicate the need for concerted collaborative research and policy endeavours directed towards improving the dissemination and uptake of evidence into practice.

## Supporting Information

Appendix S1 **Vignettes for the acute and chronic presentations of shoulder pain.**
(DOCX)

Appendix S2 **Reasonable management and rationale based upon the authors’ synthesis of the best available evidence for each vignette.**
(DOCX)
