# Supplementary material for: General Practitioner Management of Shoulder Pain in Comparison with Rheumatologist Expectation of Care and Best Evidence: An Australian National Survey
Source: PLoS One. 2013 Apr 16;8(4):e61243. doi: 10.1371/journal.pone.0061243 (PMC3628939; doi:10.1371/journal.pone.0061243)
Supplement: Appendix S1 — Vignettes for the acute and chronic presentations of shoulder pain. (DOCX) [file pone.0061243.s001.docx]

| **Appendix S1: Vignettes for the acute and chronic presentations of shoulder pain** |
| --- |
| **Vignette 1**  A 77-year-old woman, a retired bookkeeper living with her husband, presents with a 6-week history of discomfort in her right shoulder/deltoid region while sleeping, and difficulty doing her hair, putting on her coat, doing up her bra and reaching up to high shelves. On examination there is tenderness over the lateral aspect of the shoulder and pain on shoulder abduction in the mid-range but a normal range of movement. The remainder of the findings on physical examination are normal. There is no history of trauma. She has been previously well with no history of serious illness. A previous GP prescribed a 2-week course of NSAID, which didn’t provide any relief. |
| **Vignette 2**  A 45 year-old labourer sustained a work-related injury to his non-dominant left shoulder two weeks ago. A 100kg door he was carrying slipped from his grip and he felt a sharp pain in his shoulder as he attempted to stop the door from falling. His foreman made him go to the hospital where x-rays of his shoulder were normal. Since then he reports that his pain is still present, but has improved. However, he has been unable to return to work because he cannot raise his arm above his chest height. |
| **Vignette 3**  A 50 year old, right-hand-dominant female executive presents with a 3-week history of pain and progressive loss of motion of her left shoulder without history of trauma. The pain has been severe and interfering with sleep. On physical exam, a global loss of active and passive range of motion is noted with forward elevation to 45 degrees, internal rotation to the sacrum and external rotation to 10 degrees. |
| **Vignette 4**  You review the woman from the previous scenario two months later. Her pain is somewhat improved but she still has a very stiff shoulder and on physical examination there is still a 50% global loss of active and passive range of motion in all directions. |
