# Supplementary material for: General Practitioner Management of Shoulder Pain in Comparison with Rheumatologist Expectation of Care and Best Evidence: An Australian National Survey
Source: PLoS One. 2013 Apr 16;8(4):e61243. doi: 10.1371/journal.pone.0061243 (PMC3628939; doi:10.1371/journal.pone.0061243)
Supplement: Appendix S2 — Reasonable management and rationale based upon the authors’ synthesis of the best available evidence for each vignette. (DOCX) [file pone.0061243.s002.docx]

**Appendix S2 Reasonable management and rationale based upon the authors’ synthesis of the best available evidence for each vignette**

| **Management** | **Rationale** |
| --- | --- |
| **VIGNETTE 1** | |
| In the absence of red flags, neither plain X-Rays nor ultrasound likely to be helpful | Plain radiographs may be useful to look for suspected glenohumeral osteoarthritis, calcific deposits (if pain onset is rapid), large subacromial spurs (if clinical features of significant impingement), significant elevation of the humeral head and narrowing of the subacromial space (if a large rotator cuff tear is suspected)([1](#_ENREF_1)). In this vignette the pain had been present for six weeks making acute calcific tendonitis unlikely; and there was a normal range of movement making clinically significant glenohumeral osteoarthritis or a large rotator cuff tear also highly unlikely. In a 77-year old female changes in the rotator cuff on ultrasound examination would be expected so it is not clear how the findings would influence management. |
| Advice on activity and work modification; paracetamol and/or oral NSAIDs or stronger analgesia for short-term use if pain relief is insufficient ([1](#_ENREF_1)) | Many treatments have been advocated to treat rotator cuff disease however evidence from high quality trials is limited and most interventions are still of unknown effectiveness ([2](#_ENREF_2)). For example, while there is some evidence that oral NSAIDs provide short-term pain relief, there are no randomised controlled trials for topical NSAIDs, paracetamol or opioids ([2](#_ENREF_2)). In this vignette, further NSAIDs are questionable in view of lack of previous response and advanced age ([3](#_ENREF_3)). |
| Subacromial glucocorticoid injection | Subacromial injection of glucocorticoid provides rapid although short-lived pain relief ([4](#_ENREF_4), [5](#_ENREF_5)). A recent Cochrane review that included five trials and 290 participants did not find that ultrasound-guided injection provided any additional benefits over anatomic landmark-guided injection or systemic injection for shoulder pain ([6](#_ENREF_6)).In view of the added costs and lack of evidence of added benefit, the justification for routine referral for ultrasound-guided glucocorticoid injection for rotator cuff disease remains unclear. |
| Physiotherapy referral | There is some evidence that manual therapy combined with strengthening exercises improve shoulder strength and function but the benefits may not be immediately apparent ([7](#_ENREF_7)). |
| Prognosis | Follow up studies of people with shoulder pain in primary care (the majority of whom are likely to have rotator cuff disease), have reported that one in four to five new episodes of shoulder pain are resolved within 4 to 6 weeks although over half are still not resolved by a year to 18 months ([8](#_ENREF_8), [9](#_ENREF_9)) and recurrences are common ([10](#_ENREF_10)). Short duration of symptoms at presentation is associated with a better outcome than presentation with chronic symptoms ([11](#_ENREF_11), [12](#_ENREF_12)) and two studies with 3-year and 10-year follow up respectively ([13](#_ENREF_13), [14](#_ENREF_14)), found that about 50% of people presenting to primary care with new complaints of shoulder pain consulted their GP only once. |
| Surgery only for people with persistent symptoms ([1](#_ENREF_1), [15](#_ENREF_15)). | A Cochrane review was unable to draw firm conclusions about the benefits of surgery for rotator cuff disease but three trials demonstrated similar outcomes from arthroscopic or open subacromial decompression and an active rehabilitation program that includes exercise ([16](#_ENREF_16)). |
| **VIGNETTE 2** | |
| Ultrasound or MRI reasonable options | Inability to lift the arm above the shoulder in the setting of an acute injury suggests an acute rotator cuff tear. A *repeat* X-ray is unlikely to yield new information. Ultrasound or MRI are reasonable options to detect presence and severity of an acute rotator cuff tear ([17](#_ENREF_17)). |
| Refer to an orthopaedic surgeon. Anti-depressants are not indicated. | While there is a paucity of data to guide management of acute rotator cuff tear with no trials having evaluated the role of surgery compared with conservative treatment or early versus late surgery, early repair of acute rotator cuff tears may lessen the risk of chronic tendon and muscle pathology and improve functional outcomes ([18](#_ENREF_18)). While anti-depressants are widely used to treat chronic painful musculoskeletal conditions ([19](#_ENREF_19)), it is unclear what the rationale, if any, would be for acute pain. |
| **VIGNETTES 3 and 4** | |
| No imaging is necessary. | A thickened coracohumeral ligament on ultrasound might suggest adhesive capsulitis ([20](#_ENREF_20)), but most cases of adhesive capsulitis can be diagnosed clinically. Global restriction of shoulder movement, especially external rotation is a characteristic feature. Ultrasound findings might increase diagnostic confusion as approximately 50% of women of this age are expected to have rotator cuff abnormalities([21](#_ENREF_21)). |
| Intra-articular glucocorticoid injection; short course of oral glucocorticoids, arthrographic distension with combination of glucocorticoid and saline. | A Cochrane review found that intra-articular glucocorticoid injection rapidly relieves pain in people with adhesive capsulitis, although benefits may be short lived([1](#_ENREF_1), [4](#_ENREF_4), [22](#_ENREF_22)). A Cochrane review found that a short course of oral glucocorticoids provides worthwhile short-term benefits ([1](#_ENREF_1), [23](#_ENREF_23)). Arthrographic distension of the glenohumeral joint with glucocorticoid and saline (hydrodilatation) also may provide sustained benefits ([24](#_ENREF_24)), although it may be poorly tolerated in the early painful phase of adhesive capsulitis. |
| Home exercises, physical exercise program. | Supervised exercises may produce sustained improvements in shoulder mobility and function when used in combination with either glucocorticoid injection or arthrographic distension([25](#_ENREF_25), [26](#_ENREF_26)) although its cost-effectiveness is unproven ([26](#_ENREF_26)). |
| Recovery is likely or very likely to occur within 1-2 years. | Adhesive capsulitis has a favourable clinical course ([27-29](#_ENREF_27)). Although published retrospective and prospective studies have generally found that mild persistent symptoms occur in a minority, significant long-term disability is uncommon ([27-30](#_ENREF_27)). Recurrences involving the same shoulder have not been reported. |

**APPENDIX S2 REFERENCES**

1. Expert Rheumatology Group. (2010) Therapeutic guidelines: rheumatology. version 2 Melbourne: Therapeutic Guidelines Limited.

2. Murphy RJ, Carr AJ. (2010) Shoulder pain. Clin Evid (Online) pii: 1107.

3. Glazier RH, Dalby DM, Badley EM, Hawker GA, Bell MJ, et al. (1998) Management of common musculoskeletal problems: a survey of Ontario primary care physicians. Can Med Assoc J 158: 1037-1040.

4. Buchbinder R, Green S, Youd JM. (2003) Corticosteroid injections for shoulder pain. Cochrane Database System Rev 1: CD004016.

5. Arroll B, Goodyear-Smith F. (2005) Corticosteroid injections for painful shoulder: a meta-analysis. Br J Gen Pract 55:224-228.

6. Bloom J, Rischin A, Johnston R, Buchbinder R. (2012) Image-guided versus blind glucocorticoid injection for shoulder pain Cochrane Database System Rev 8: CD009147.

7. Bennell K, Wee E, Coburn S, Green S, Harris A, et al. (2010) Efficacy of standardised manual therapy and home exercise programme for chronic rotator cuff disease: randomised placebo controlled trial. Brit Med J 340: c2756.

8. van der Windt DA, Koes BW, Boeke AJ, Deville W, De Jong BA, et al. (1996( Shoulder disorders in general practice: prognostic indicators of outcome. Br J Gen Pract 46: 519-523.

9. Croft P, Pope D, Silman A. (1996) The clinical course of shoulder pain: prospective cohort study in primary care. Primary Care Rheumatology Society Shoulder Study Group. Brit Med J 313: 601-602.

10. Winters JC, Sobel JS, Groenier KH, Arendzen JH, Meyboom-de Jong B. (1999) The long-term course of shoulder complaints: a prospective study in general practice. Rheumatol 38: 160-163.

11. Thomas E, van der Windt DA, Smidt N, Dziedzic K, et al. (2005) Two pragmatic trials of treatment for shoulder disorders in primary care: generalisability, course, and prognostic indicators. Ann Rheum Dis 64: 1056-1061.

12. Reilingh M, Kuijpers T, Tanja-Harfterkamp A, van der Windt DA. (2008) Course and prognosis of shoulder symptoms in general practice. Rheumatol 47: 724-730.

13. Linsell L, Dawson J, Zondervan K, Rose P, Randall T, et al. (2006) Prevalence and incidence of adults consulting for shoulder conditions in UK primary care; patterns of diagnosis and referral. Rheumatol 45: 215-221.

14. Dorrestijn O, Greving K, van der Veen W, van der Meer K, Diercks R, et al. (2011) Patients with shoulder complaints in general practice: consumption of medical care. Rheumatol 50: 389-395.

15. American Academy of Orthopaedic Surgeons. (2010) American Academy of Orthopaedic Surgeons Clinical Practice Guideline on optimizing the management of rotator cuff problems: Guidelines and evidence report. Rosemont, IL: American Academy of Orthopaedic Surgeons.

16. Coghlan JA, Buchbinder R, Green S, Johnston R, Bell S. (2008) Surgery for rotator cuff disease. Cochrane Database System Rev 1: CD005619.

17. Dinnes J, Loveman E, McIntyre L, Waugh N. (2003) The effectiveness of diagnostic tests for the assessment of shoulder pain due to soft tissue disorders: a systematic review. Health Technol Assess 7: 1-166.

18. American Academy of Orthopaedic Surgeons. (1999) Clinical Guideline on Shoulder Pain. Rosemont, IL: American Academy of Orthopaedic Surgeons.

19. Perrot S, Maheu E, Javier R, Eschalier A, Coutaux A, et al. (2006) Guidelines for the use of antidepressants in painful rheumatic conditions. Eur J Pain 10: 185-192.

20. Homsi C, Bordalo-Rodrigues M, da Silva JJ, Stump XM. (2006) Ultrasound in adhesive capsulitis of the shoulder: is assessment of the coracohumeral ligament a valuable diagnostic tool? Skel Radiol 35: 673-678.

21. Milgrom C, Schaffler M, Gilbert S, van Holsbeeck M. (1995) Rotator-cuff changes in asymptomatic adults. The effect of age, hand dominance and gender. J Bone Joint Surg Br 77: 296-298.

22. Favejee M, Huisstede B, Koes B. (2010) Frozen shoulder: the effectiveness of conservative and surgical interventions— systematic review. Br J Sports Med 45: 49-56.

23. Buchbinder R, Green S, Youd JM, Johnston RV. (2006) Oral steroids for adhesive capsulitis. Cochrane Database System Rev 4: CD006189.

24. Buchbinder R, Green S, Youd JM, Johnston R, Cumpston M. (2008) Arthrographic distension for adhesive capsulitis (frozen shoulder) Cochrane Database of System Rev 1: CD007005.

25. Carette S, Moffet H, Tardif J, Bessette L, Morin F, et al. (2003) Intraarticular corticosteroids, supervised physiotherapy, or a combination of the two in the treatment of adhesive capsulitis of the shoulder: a placebo-controlled trial. Arthritis Rheum 48: 829-838.

26. Buchbinder R, Youd J, Green S, Stein A, Forbes A, et al. (2007) Efficacy and cost-effectiveness of physiotherapy following glenohumeral joint distension for adhesive capsulitis: A randomized trial. Arthritis Care Res 57: 1027-1037.

27. Reeves B. (1975) The natural history of the frozen shoulder syndrome. Scand J Rheumatol 4: 193-196.

28. Binder AI, Bulgen DY, Hazleman BL, Roberts S. (1984) Frozen shoulder: a long-term prospective study. Ann Rheum Dis 43: 361-364.

29. Hand C, Clipsham K, Rees JL, Carr AJ. (2008) Long-term outcome of frozen shoulder. J Shoulder Elbow Surg 17: 231-236.

30. Vastamaki H, Kettunen J, Vastamaki M. (2012) The natural history of idiopathic frozen shoulder: a 2- to 27-year followup study. Clin Orthop Rel Res 470: 1133-1143.
